# Freeze-Drying Processes Applied to Melon Peel: Assessment of Physicochemical Attributes and Intrinsic Microflora Survival during Storage

**DOI:** 10.3390/foods11101499

**Published:** 2022-05-21

**Authors:** Sengly Sroy, Fátima A. Miller, Joana F. Fundo, Cristina L. M. Silva, Teresa R. S. Brandão

**Affiliations:** CBQF-Centro de Biotecnologia e Química Fina–Laboratório Associado, Escola Superior de Biotecnologia, Universidade Católica Portuguesa, Rua Diogo Botelho 1327, 4169-005 Porto, Portugal; sengly.sroy@gmail.com (S.S.); jfundo@ucp.pt (J.F.F.); clsilva@ucp.pt (C.L.M.S.)

**Keywords:** ozone pre-treatment, bioactive compounds, antioxidant activity, total mesophylls, molds and yeasts

## Abstract

Melon peel is recognized as a source of healthy nutrients and oxidant compounds. Being considered a non-edible part with no profit value, large amounts of melon rinds are discharged by fruit industries. Innovative food ingredients with potential health benefits may arise if these parts were conveniently transformed. The objective was to freeze-dry small melon peel cubes to attain a potential edible matrix. An ozone pre-treatment was applied seeking decontamination purposes and quality retention. The effect of these processes was assessed in terms of physicochemical parameters (moisture content, water activity and color), bioactive compounds (total phenolics, vitamin C and chlorophylls) and antioxidant capacity, during 7 weeks of storage at room temperature. Intrinsic microflora (mesophylls, yeasts and molds) were also monitored. Results showed that the freeze-drying process allowed retention of the most bioactive compounds analyzed, except for total phenolic content. In this case, the ozone pre-treatment was important for phenolics preservation. During the storage period, ozonated samples presented a higher content of bioactive compounds. In terms of microflora, the ozone and freeze-drying effects were not significant. Freeze-drying proved to be a suitable preservation method for melon peel. The ozone impact was not relevant in terms of decontamination.

## 1. Introduction

Fruit industries produce waste quantities on a large scale. In the particular case of melon, significant amounts of peel are discharged as residues after juice processing. This is critical from an environmental and economic point of view. Since these wastes are considered rich sources of bioactive compounds, their transformation and reuse are extremely important [1,2]. A study on the characterization of melon (*Cucumis melo* L. var. *reticulatus*) showed that the peel contains 32, 30, 27 and 15% of all total phenolics, potassium, antioxidant activity and total carotenoids content, respectively [3]. Therefore, developing new strategies to turn this non-edible part into a valuable and attractive ingredient is of utmost interest [4,5]. Several works have been developed to valorize fruit wastes in the last decade to produce novel nutraceuticals, pharmaceuticals, cosmeceuticals, food ingredients or functional foods. As additives in food products, fruit wastes have been used to preserve and enhance food quality [6], as a supply of bioactive compounds [7], as an encapsulating agent [8], to produce edible packaging material [9] and also in the production of food products with enhanced prebiotic activity [10]. Fruit wastes are also used as biofertilizers [11], in pharma drugs, as a bio-adsorbent for heavy metals removal [12], in biofuel production [13] and as an additive for some skincare products.

Drying is a well-studied process used in the food industry to extend products’ self-life by inhibiting microorganisms’ growth and enzyme activity [14]. Dried food products are considered safe since their water activity (a_w_) is lower than 0.80, a value at which most bacteria cannot grow. Nevertheless, since yeasts and molds are more tolerant to lower a_w_ values, special attention should be given to this group of microorganisms when drying food products [15].

Among drying processes, freeze-drying is recognized as the one that allows better retention of overall food quality characteristics [2,16]; shape, dimension, appearance, taste, color and flavor are examples. The freeze-dried product is usually highly porous, brittle, hygroscopic and has excellent rehydration capacity [14]. Nevertheless, some sensory and nutritional parameters can be negatively affected.

When conveniently packaged, a dried product has an extended shelf-life and can be considered a suitable transformation of raw materials that can be incorporated into various food matrices. In the particular case of fruit wastes such as melon rinds, drying small pieces of the product or grinding it to attain powders with adequate granulometry may open opportunities to develop new food ingredients while reducing wastes and contributing to a circular economy.

Raw foods, particularly fruits and vegetables, are grown near the soil and their surfaces can be heavily contaminated with microorganisms. Therefore, decontamination treatments are essential to guarantee a safe product for human consumption. Treatments that avoid the negative impact of high temperatures on the quality features of food products are often applied. Ozone is gaining importance in this field, with broad applications in fruit processing. Exposing food products to ozone may allow the inactivation of undesirable microorganisms with positive effects on several bioactive compounds, which are beneficial to health [17,18].

The main objective of this work was to assess the impact of the freeze-drying process on melon peel cubes with and without ozone pre-treatment. Another important goal was to evaluate the effect of these processes during 7 weeks of storage at room temperature. Physicochemical characteristics (moisture content, a_w_ and color), bioactive compounds (total phenolics, vitamin C and total chlorophylls) and antioxidant capacity were assessed weekly. Microbial indicators (total mesophylls and yeasts and molds) were also monitored during the storing period.

Raw melon rinds are non-edible wastes, but a value-added ingredient may be created if conveniently processed and transformed.

## 2. Materials and Methods

### 2.1. Sample Preparation

Cantaloupe melons (*Cucumis melo* L. var. *reticulatus*) were obtained from a local supermarket at the commercial maturity stage and stored overnight at 4 °C. Fruits selection was based on visual inspection, without physical damage, defects or disease. Using a sharp stainless-steel knife, the melon peel was cut in cubic shapes with dimensions between 0.064 to 0.216 cm^3^.

Before freeze-drying and storage, part of the melon peel underwent an ozone pre-treatment; the other part did not suffer the ozonation process. As described below, fresh-cut and processed samples were analyzed in terms of physicochemical characteristics, bioactive compounds, antioxidant activity and microbial indicators.

### 2.2. Ozone Pre-Treatment

Ozone gas (O_3_) was generated with a corona discharge equipment model (OZ5, SPO3-Sociedade Portuguesa de Ozono, Lda. Porto, Portugal), producing ozone at 5 g/h. Pure oxygen was supplied via an oxygen cylinder at 0.5 bar. Ozone was continuously bubbled for 30 min into an airtight plastic box (12 cm × 27 cm × 17 cm) with a thin film of 300 g of peel cubes, as described by Miller et al. [18]. The corresponding ozone dose attained was 152 ± 71 ppm at a controlled temperature of 15 °C. This treatment time was chosen since previous studies reported more than 1 log cycle reduction of *Listeria innocua* [18]. Each treatment was carried out three times, independently.

### 2.3. Freeze-Drying Process

Fresh and ozonized peels (50.0 g) were packed in sterilized plastic flasks (100.0 mL), frozen at −80 °C for 6 to 8 h and then transferred to a Heto Drywinner freeze-dryer (Cambridge Biosystems, Cambridge, United Kingdom). Samples were subjected to a temperature of −50 °C and a pressure ranging from 1.5 to 2 bar, for 80 to 90 h. Three replicates of the drying process were carried out.

### 2.4. Storage

Dried samples, placed in sterilized plastic flasks (100.0 mL), were kept in the dark, at room temperature, for 7 weeks.

### 2.5. Physicochemical Analysis

#### 2.5.1. Moisture Content

The moisture content of melon peel was determined according to the oven drying method recommended by the Association of Analytical Chemistry (Method 984.25; AOAC 2002 [19]). Briefly, approximately 10 g of melon peel samples were dehydrated in an air oven (Memmert, Labmetro, Germany) at 105 °C until no weight alteration was detected. Weight measurements were carried out in an analytical balance (Mettler Toledo AE 200, Marshall Scientific, Knutsford, UK) with a precision of ± 0.0001 g. Results were expressed as the difference between fresh and dried peel weights, divided by the fresh wet weight (expressed in %). Moisture content was determined in triplicate.

#### 2.5.2. Water Activity

For water activity (a_w_*)* measurements, a dew point hygrometer (Aqualab–Series 3, Decagon Devices Inc., USA) was used at 22 ± 1 °C. Two readings of three replicated samples were carried out.

#### 2.5.3. Color

CIELAB color components of peel samples (L*, a*, b*) were measured using a Minolta CR-400 colorimeter (Konica-Minolta, Osaka, Japan). Total color difference (TCD) was estimated according to Equation (1), which accounts for color changes between fresh and processed samples [20,21]. Higher TCD values indicate more noticeable color alterations.

(1) $$\mathrm{TCD}=\sqrt{{\left(L_{0}^{*}-L^{*}\right)}^{2}+{\left(a_{0}^{*}-a^{*}\right)}^{2}+{\left(b_{0}^{*}-b^{*}\right)}^{2}}$$

The index “0” corresponds to the fresh melon peel samples. Two readings of three different replicates were conducted for each sample.

### 2.6. Bioactive Compounds

#### 2.6.1. Total Phenolic Content

The total phenolic content of peel samples was determined according to the Folin–Ciocalteu method using gallic acid as standard [5]. Extractions were done by mixing 25.0 g of the peel with 50.0 mL of 100% methanol (Merck) [18]. The results were expressed in gallic acid equivalent (GAE) in μg/g of dried melon peel.

#### 2.6.2. Vitamin C

Ascorbic acid (AA) and dehydroascorbic acid (DHA) were determined following the HPLC analytical procedure outlined by Zapata and Dufour [22]. A reverse phase C18-silica analytical column (Waters Spherisorb ODS2 5 µm 4.6 × 250 mm) was used, and mobile phase, standard solutions, and samples were prepared as described by Fundo et al. [3]. Results were reported as mg of vitamin C per 100 g of dried peel.

#### 2.6.3. Chlorophylls

Chlorophylls were extracted, homogenizing 7.0 g of the melon peel with 50.0 mL of 100% methanol with an ultra-turrax. Extracts were then centrifuged at 5000× *g* at 4 °C for 10 min and filtered using filter paper. The extract absorbance was measured at given wavelengths and the quantification of chlorophylls a and b (µg/mL) was based on Equations (2) and (3) [23]. Total chlorophylls is the sum of chlorophylls a and b.

(2) $$\mathrm{Chlorophyll}\;a=16.72{\;A}_{665.2\mathrm{nm}}-9.16{\;A}_{652.4\mathrm{nm}}$$

(3) $$\mathrm{Chlorophyll}\;b=34.09{\;A}_{652.4\mathrm{nm}}-15.28{\;A}_{665.2\mathrm{nm}}$$

Results were expressed in µg of chlorophylls per gram of dried peel.

### 2.7. Antioxidant Activity

Total antioxidant activity was estimated using the ABTS assay, according to Fundo et al. [3]. The results were expressed as μg of AA per gram of dried peel.

### 2.8. Microbiological Analysis

Melon peel samples (0.5 g) were homogenized with 49.5 mL of buffered peptone water (BPW) in a stomacher for 2 min. Decimal dilutions were carried out in BPW. Total mesophylls were assessed in duplicate using Plate Count Agar–PCA (Lab M, Lancashire, UK). Plates were incubated at 37 °C for 48 h. Molds and yeasts were evaluated in duplicate using Rose Bengal Agar-RBA (Lab M, Lancashire, UK), with the plates incubated at 25 °C for 60 h. Results were expressed as log_10_ CFU per gram of dried peel.

### 2.9. Statistical Analysis

Differences between samples were analysed by one-way ANOVA concerning all characteristics studied. Means were compared by Tukey’s test, while normality and homoscedasticity of data were assessed using Shapiro–Wilk and Levene’s tests, respectively. When data normality was not proved, the Kruskal–Wallis test was carried out alternatively to one-way ANOVA. In such cases, the non-parametric Mann–Whitney test was used to detect differences. Data analyses were performed at a significance level of 5% using IBM SPSS Statistics 24 for Windows^®^ (SPSS Inc., Chicago, IL, USA). The results were reported as a mean ± margin of confidence interval at 95%. The confidence interval margin is half of the confidence interval at 95%.

## 3. Results and Discussion

### 3.1. Effect of Ozone Pre-Treatment on Dried Samples

#### 3.1.1. Physicochemical Characteristics

The effect of ozone pre-treatment on moisture content, a_w_ and color of dried melon peel can be assessed from the data presented in Table 1. After the drying process, and as expected, moisture content strongly decreased (approximately 88%). The effect of ozone exposure was not evident, with no significant differences observed between ozonized and non-ozonized samples. Akbas and Ozdemir [24] also reported no differences in moisture content of ozonized and non-ozonized figs after drying. Contrarily, the ozone process significantly decreased a_w_ of dried peel samples compared with non-ozonized ones. Based on these results, it is possible to speculate that although ozone exposure did not change the amount of water present in the samples, it decreased the available water for microorganisms’ growth and chemical reactions. This is probably due to the molecular interactions between free water and O_3_ constituents.

Color alterations frequently occur in dried food matrixes due to non-enzymatic browning reactions [14]. Both non-ozonized and ozonized dried peel samples presented significantly higher L* values, indicating of a brighter color when compared to fresh peel [25]. Dried samples’ greenness (a*) and yellowness (b*) significantly changed. However, no significant differences were observed between non-ozonized and ozonized dried peels on the three color coordinates. The values of TCD were higher than 12 units for both dried and ozonated dried melon peel. According to Drlange [26], this can be considered a very great perceived color difference when compared to fresh samples. Fernandes et al. [27] also attained similar results for freeze-dried pumpkins.

#### 3.1.2. Bioactive Compounds and Oxidant Activity

The results of total phenolic content, vitamin C, total chlorophylls and antioxidant activity are included in Table 2 for fresh, dried and ozonated dried samples.

Total phenolics were significantly reduced after the drying process of the non-ozonized peel. Ozone exposure had a positive effect on the retention of these compounds. The drying process might accelerate the release of more bound phenolic compounds due to the breakdown of cellular constituents [28]. The observed ozone positive effect can be attributed to the activation of phenylalanine ammonialyase, one of the key enzymes used to synthesize phenolic compounds in plant tissues. The total phenolic content maintenance might have been caused by peel cell wall modification during ozone exposure [29].

Vitamin C was not affected by the freeze-dried process or ozone exposure. The temperatures involved in the freeze-dried process were not high (around 50 °C), which may not affect the degradation of this water-soluble vitamin [30].

Total chlorophylls were also preserved. This can be explained by low enzymatic activity caused by the processes applied [30].

Antioxidant activity is due to the presence of different bioactive compounds, which were not affected by the freeze-drying processes. The total antioxidant activity of non-ozonized and ozonized dried peel was equivalent to fresh samples.

#### 3.1.3. Intrinsic Microorganisms

The effect of freeze drying on the intrinsic microflora of ozonized and non-ozonized peel is presented in Table 3. No significant differences were observed in total mesophylls, molds and yeasts of fresh and dried samples.

Since freeze-drying decreased peel a_w_ to values lower than 0.80, in which most bacteria cannot grow, shelf-life studies are essential. The ozone impact was not relevant, being that the microorganisms log counts of dried samples, with or without O_3_ pre-treatment, were equivalent to the ones enumerated in the fresh melon peel. Although several fruits had similar results, a slight decrease in yeasts and molds counts was observed, after 1 h of ozone treatments [17,31].

### 3.2. Shelf-Life Assessment of Non-Ozonized and Ozonized Dried Peel

#### 3.2.1. Physicochemical Characteristics

**Figure 1:**
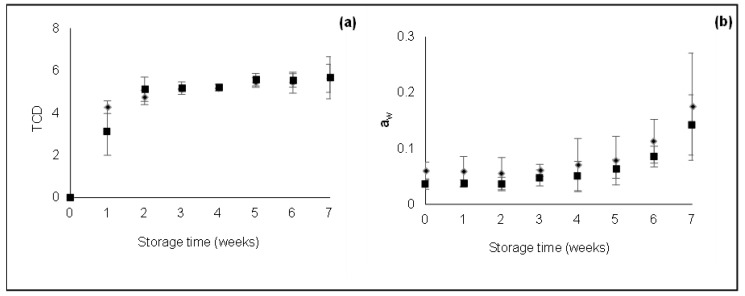
TCD (**a**) and a_w_ (**b**) of non-ozonized (◆) and ozonized (■) dried peels during storage. Data represent mean values and bars represent the limits of confidence intervals at 95%.

Total color difference and a_w_ of non-ozonized and ozonized dried peel during 7 weeks of storage are shown in Figure 1.

The TCD references (index 0 in Equation (1)) were the color coordinates obtained for dried samples. As shown in Figure 1a, TCD increased in the first two weeks of storage. After this period, TCD remained constant until the end of storage time. Similar to these results, Topuz et al. [32] detected dried paprika color alterations in the first 30 days of storage, with no significant differences attained after that period. No significant differences were observed between non-ozonized and ozonized samples.

The a_w_ of non-ozonized and ozonized dried peel during the storage is shown in Figure 1b. During the storage period, no significant differences were observed between ozonized and non-ozonized samples. However, after 5 weeks of being stored, the a_w_ of the dried peel slightly increased. The fact that samples were stored without humidity controlling conditions may explain this increase. This must be taken into consideration when longer storage periods are imposed.

#### 3.2.2. Bioactive Compounds and Antioxidant Activity

**Figure 2:**
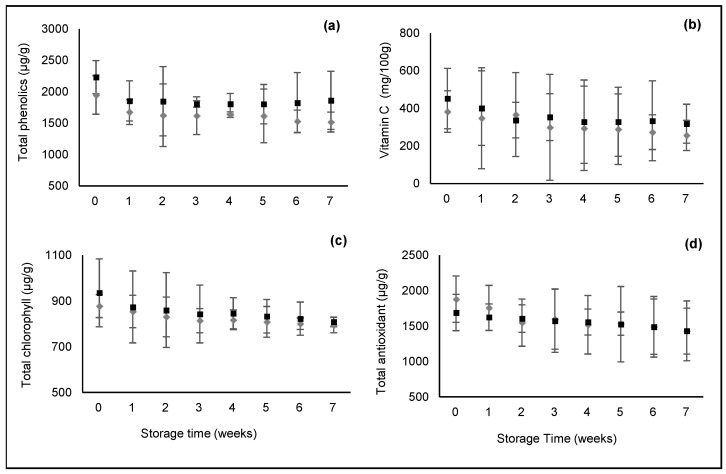
Total phenolic content (**a**) vitamin C, (**b**) total chlorophylls content and (**c**) total antioxidant activity (**d**) in non-ozonized (◆) and ozonized (■) dried peel. Data represent mean values and bars represent the limits of confidence intervals at 95%.

The behavior of the bioactive compounds and total antioxidant activity of non-ozonized and ozonized dried peel during storage are presented in Figure 2. As observed in Figure 2a, the total phenolic content of non-ozonized and ozonized dried samples decreased during the storage period. However, samples with O_3_ pre-treatment retained better total phenolics throughout the entire storage period. Tzortzakis et al. [33] reported that ozone exposure increased total phenolic content in tomatoes.

Vitamin C, in Figure 2b, also decreased during the storage period for both non-ozonized and ozonized dried samples, being slightly higher in ozonated ones. Vitamin C is very susceptible to chemical and enzymatic oxidation during food processing and storage. According to Laing et al. [34], ascorbic acid degradation rates depend on oxygen availability, temperature and moisture content, and the higher the water activity, the greater the vitamin C loss. The increase of a_w_ during the storage period may also explain the occurrence of vitamin C losses.

The green color of fruits peel is mainly due to chlorophylls, which are highly susceptible to degradation during processing and storage [35]. The most abundant type of chlorophyll in melon peel is chlorophyll a (around 530 µg/g), followed by chlorophyll b (about 400 µg/g). Both types of chlorophylls showed a similar slightly degradation during the 7 weeks of storage (both non-ozonized and ozonized dried peels; results not shown). In Figure 2c, it is possible to see the effect of storage time on the total chlorophylls of dried samples. As previously mentioned, ozone may inhibit the enzymes responsible for chlorophyll degradation through the induction of antioxidants that can protect chlorophylls [36]. Although ozonized dried samples seem to better retain these compounds during storage, the difference was not significant. At the end of storage, the value for both ozonized and non-ozonized peels is almost the same.

In terms of total antioxidant activity, differences between ozonated and non-ozonated samples are not significant, as shown in Figure 2d. Both samples presented a similar tendency during the entire storage period. Since the antioxidant activity of fruits may be attributed to the presence of vitamin C, polyphenols and other bioactive compounds, the observed decrease was expected.

#### 3.2.3. Intrinsic Microflora

Regarding total mesophylls counts and at the end of the storage period, 1.40 ± 0.52 and 0.90 ± 1.22 log cycles were reduced in non-ozonized and ozonized samples, respectively. For yeasts and molds, 0.85 ± 1.02 and 1.00 ± 0.48 log cycles reduction were obtained for non-ozonized and ozonized dried peels, respectively. The effect of O_3_ was not significant in the microflora analyzed.

Total mesophylls, yeasts and molds were the microorganisms chosen as indicators of the drying process’s effectiveness and ozone treatment during storage. For both groups of microorganisms, freeze-drying showed to be effective in controlling microbial growth during the 7 weeks of storage. Freeze-drying at low temperatures may stop microbiological activity, providing food products with better quality [15].

Despite the well-known effect of ozone on microbial inactivation by the progressive oxidation of vital cell components, preventing microbial growth and extending the shelf-life of many fruits [37], this effect was not observed since no significant differences were attained on microbial loads of non-ozonized and ozonized dried peels.

## 4. Conclusions

Freeze-drying of melon peel proved to be effective in preserving of the bioactive compounds analyzed, except for total phenolic content. Shelf-life studies of dried samples revealed that pre-ozonized samples better retained total phenolics, vitamin C and total chlorophylls.

In terms of microflora inactivation, the effects of ozone and freeze-drying were not significant. However, at the end of storage, a decrease of around 1 log-cycle was observed for the groups of microorganisms studied. Melon rinds exposed to gaseous ozone did not achieve convenient decontamination. Further studies with undesirable target microorganisms are required to attain a safe product.

Freeze-drying can be considered a potential process to transform melon peel into an edible form. When the small cubes of melon rinds were freeze-dried, they became lighter and softer. Grinding these dried materials will also allow for transformation into powders. They can be incorporated into different food products, such as cakes, breads and yogurts, enriching their nutritional value. Moreover, the dried material itself may constitute a novel food ingredient: gluten free flours with a wide range of applications in the food industry, for example. However, a deep study about the toxicity of melon peel compounds should be attained before consuming the products.

## Figures and Tables

**Table 1:** Selected physicochemical characteristics of fresh and freeze-dried melon peel, with and without ozone pre-treatment. The values are mean ± margin of confidence intervals at 95%.

Physicochemical Characteristic		Melon Peel		
		Fresh	Dried	Ozonated + Dried
moisture content (%)		87.51 ± 6.79 ^b^	12.37 ± 2.79 ^a^	12.41 ± 2.46 ^a^
a_w_		0.99 ± 0.0092 ^c^	0.06 ± 0.02 ^b^	0.04 ± 0.01 ^a^
color	L* a*	47.29 ± 3.24 ^a^ −12.79 ± 1.31 ^a^	66.00 ± 4.24 ^b^ −4.82 ± 1.98 ^b^	63.73 ± 3.61 ^b^ −4.69 ± 2.87 ^b^
	b*	33.21 ± 2.94 ^b^	24.65 ± 2.42 ^a^	21.56 ± 1.49 ^a^
	TCD	-	22.11 ± 10.99 ^a^	21.12 ± 9.39 ^a^

For a given characteristic, values with different letters differ significantly (*p* < 0.05).

**Table 2:** Bioactive compounds and total antioxidant activity of fresh and freeze-dried melon peels, with and without ozone pre-treatment. The values are mean ± margin of confidence intervals at 95%.

Compound	Fresh	Dried	Ozonated + Dried
Total phenolic content (μg/g)	2447.54 ± 241.23 ^b^	1948.91 ± 307.01 ^a^	2232.60 ± 262.49 ^ab^
Vitamin C (mg/100g)	423.61.7 ± 236.32 ^a^	383.27 ± 110.85 ^a^	452.56 ± 160.18 ^a^
Total chlorophylls (μg/g)	941.61 ± 85.59 ^a^	877.93 ± 50.01 ^a^	935.67 ± 148.62 ^a^
Total antioxidant activity (μg/g)	1806.97 ± 236.97 ^a^	1879.83 ± 327.58 ^a^	1690.80 ± 255.43 ^a^

For a given characteristic, values with different letters differ significantly (*p* < 0.05).

**Table 3:** Effect of freeze-drying on the intrinsic microflora of fresh and ozonated melon peel. The values are mean ± margin of confidence intervals at 95%.

Microflora	Fresh	Dried	Ozonated + Dried
Total mesophylls (Log CFU/mL)	6.48 ± 1.51 ^a^	6.26 ± 1.30 ^a^	5.45 ± 2.14 ^a^
Molds and yeasts (Log CFU/mL)	4.02 ± 2.04 ^a^	4.00 ± 1.15 ^a^	3.83 ± 1.15 ^a^

For a given characteristic, values with different letters differ significantly (*p* < 0.05).

## Data Availability

Data is contained within the article.
